# Large Particle 3D Concrete Printing—A Green and Viable Solution

**DOI:** 10.3390/ma14206125

**Published:** 2021-10-15

**Authors:** Inka Mai, Leon Brohmann, Niklas Freund, Stefan Gantner, Harald Kloft, Dirk Lowke, Norman Hack

**Affiliations:** 1 Institute of Building Materials, Concrete Construction and Fire Safety, Technische Universität Braunschweig, 38106 Braunschweig, Germany; i.mai@ibmb.tu-bs.de (I.M.); n.freund@ibmb.tu-bs.de (N.F.); d.lowke@ibmb.tu-bs.de (D.L.); 2 Institute of Structural Design, Technische Universität Braunschweig, Pockelsstr. 4, 38104 Braunschweig, Germany; leon.brohmann@tu-bs.de (L.B.); stefan.gantner@tu-bs.de (S.G.); h.kloft@tu-bs.de (H.K.)

**Keywords:** additive manufacturing in construction, 3D concrete printing, particle bed 3D printing, particle bed binding, ecology, low carbon, large particles, recycled aggregates

## Abstract

The Large Particle 3D Concrete Printing (LP3DCP) process presented in this paper is based on the particle bed 3D printing method; here, the integration of significantly larger particles (up to 36 mm) for selective binding using the shotcrete technique is presented. In the LP3DCP process, the integration of large particles, i.e., naturally coarse, crushed or recycled aggregates, reduces the cement volume fraction by more than 50% compared to structures conventionally printed with mortar. Hence, with LP3DCP, the global warming potential, the acidification potential and the total non-renewable primary energy of 3D printed structures can be reduced by approximately 30%. Additionally, the increased proportion of aggregates enables higher compressive strengths than without the coarse aggregates, ranging up to 65 MPa. This article presents fundamental material investigations on particle packing and matrix penetration as well as compressive strength tests and geometry studies. The results of this systematic investigation are presented, and the best set is applied to produce a large-scale demonstrator of one cubic meter of size and complex geometry. Moreover, the demonstrator features reinforcement and subtractive surface processing strategies. Further improvements of the LP3DCP technology as well as construction applications and architectural design potentials are discussed thereafter.

## 1. Introduction

For many years, a debate on sustainability in construction has been taking place in research and industry. Especially the environmental impact and high carbon emissions related to concrete constructions are discussed. This is due to the fact that the production of cement is inevitably accompanied by a high demand for energy and severe CO_2_ emissions, which are linked to the required chemical processes converting limestone-based raw materials into Portland cement clinker [1]. Therefore, the amount of cement is a decisive factor in determining the ecological footprint of concrete [2].

In order to make concrete construction more environmentally friendly, various approaches are feasible, including [1,2,3,4]:

- **material savings** through new design and production techniques, e.g., by producing material-efficient structures using additive manufacturing, in which material is placed only where it is structurally needed,
- **reductions in the cement content** in the concrete and,
- **(more) sustainable circular concrete composition**, for example, by adding recycled aggregates.

In this paper, additive manufacturing with cement-based materials is used equally to address the mentioned strategies and implement them into an additive manufacturing process, namely Large Particle 3D Concrete Printing (LP3DCP).

## 2. State of the Art: Basic Principles of Additive Manufacturing in Construction

Additive manufacturing—also known as 3D printing—is a core element of digital fabrication. Three-dimensional printing allows for the application of novel design principles and the intelligent and efficient utilization of materials and resources [5,6]. The customization and the freedom to create complex geometries come at no extra cost. Considering the enormous environmental impact of the construction industry (40% of global energy consumption, 38% of global greenhouse gas emissions, 12% of global potable water use and 40% of solid waste generation in developed countries) and the CO_2_ emissions due to the production of cement (5–7% of the global anthropogenic carbon emissions) [7,8,9], the implementation of 3D printing technologies in construction has the potential to have highly positive economic and ecological impacts [10,11].

Various additive manufacturing technologies for concrete are currently being developed worldwide [6,12,13,14,15,16,17,18,19,20]. In large-scale additive manufacturing in construction with cementitious materials, three main 3D printing techniques can be distinguished according to the RILEM process classification framework [21]:

(a). material extrusion,
(b). material jetting,
(c). particle-bed binding.

Further innovative techniques are currently being investigated but are still at the early stage of development, for example, Injection 3D Concrete Printing, which is overcoming the layer-by-layer build-up of the component [19,22].

Material extrusion and jetting involve the deposition of strands with extrusion [23,24] or spraying [17,25], respectively, of the premixed material through a nozzle. Here, two strategies can be distinguished: (I) the deposition of narrow strands of several centimetres width, which are either stitched to create the desired structure or used to build a filigree formwork structure strengthened by an inner structure (compare with concrete printing [26,27,28] and contour crafting [29,30,31,32]) and (II) the deposition of broad strands of several decimetres width, which are used to build the whole width of the component in a single pass as a kind of “infinite brick” (compare with, e.g., ConPrint3D [33,34]).

The major advantage of these techniques is the high manufacturing speed for creating large-sized monolithic structures. Nevertheless, these techniques have limitations with respect to freedom of form. The production of overhanging or bridging structures is possible only with restrictions (2.5 D). Resolution is usually lower than for particle-bed binding techniques.

In particle-bed binding techniques, a layer of dry particles is applied, and then a liquid phase is applied to bind the particles locally. Subsequently, the unbound particles are removed. Here, a distinction is made between two particle-bed binding processes: (I) selective paste intrusion (SPI), in which the particle bed consists of aggregates, and cement paste is selectively applied to the particle bed [35], and (II) selective cement activation [10,18] in which the particle bed consists of an aggregate-cement mixture, and the cement is locally activated by water.

Compared to the other, aforementioned additive manufacturing techniques used in construction, particle-bed binding techniques place almost no restrictions on the freedom of form. Since the dry packed particles are mechanically stable, inclined structures, overhangs, arches, vaults, suspended beams or cantilevers can easily be created [10,36]. The resolution achievable with this technique is highly dependent on the maximum aggregate size. Furthermore, production time is independent of geometric complexity. The major drawback of the particle-bed binding technique is—up to date—the limited component size due to the available printer dimensions and a lower mechanical strength compared to material extrusion or jetting. Due to the fact that larger aggregates are not included in the mixture composition, at the same time rather high cement contents are used. This is also due to the fact that no larger aggregates are included in the mixture composition. In order to overcome these limitations, in this research, large, recycled aggregates are used in the particle bed, which are selectively bound by wet mixed shotcrete. This novel process is called Large Particle 3D Concrete Printing (LP3DCP).

## 3. Large Particle 3D Concrete Printing: Basic Principle

With the selective paste intrusion process described above, strengths in the range of normal concrete up to 78 MPa were achieved so far [10,35,37,38,39]. The mechanical properties depend largely on the bond quality between the individual layers and the degree of filling of the cavities between the aggregates [35]. Therefore, rheological properties of the used cement pastes and the packing of the particle bed are particularly relevant. The average particle size in this process is <5 mm, and the selectively binding fluid is a cement paste consisting of cement, water and additives [10].

**Figure 1:**
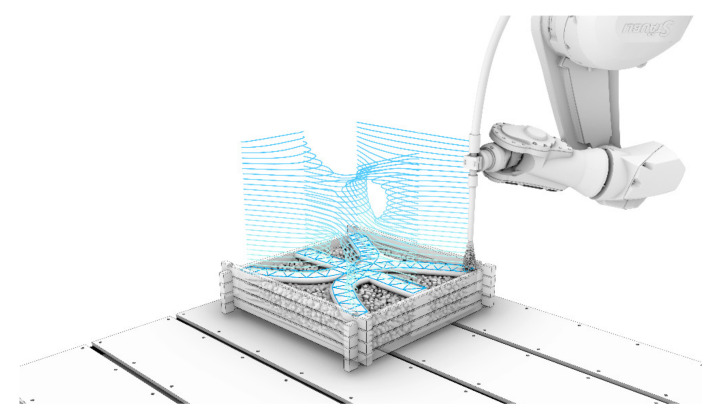
Basic concept of Large Particle 3D Concrete Printing (LP3DCP): (1) a layer of large particles, i.e., coarse aggregates, is distributed; (2) fine grain concrete is sprayed in the voids; (3) this step is repeated until the desired geometry is produced (copyrighted by the Institute for Structural Design, TU Braunschweig).

In the presented work, SPI is scaled up to significantly larger particle diameters and combined with a robot-controlled wet-mix shotcrete process. The basic idea of the LP3DCP process is shown in Figure 1. To build up the component, first, a layer of large particles, i.e., coarse aggregates, is distributed within a modular and reusable building chamber, whereby the maximum aggregate size equals the particle diameter. Secondly, fine grain concrete is selectively sprayed into the voids, whereby the amount of applied material is matched to the prevalent void content of the particle bed. Then, this step is repeated until the desired geometry is produced. Finally, the unbound dry particles are to be removed.

The LP3DCP technology allows one to reduce the amount of cement by increasing the volume and size of the aggregate. By using recycled materials, the possibility to reduce the CO_2_ footprint even further is given. Together with the material savings that can be achieved using digital production methods, building components with a significantly reduced CO_2_ footprint can be produced [6,40]. Additionally, the implementation of large particles in the process is assumed to enable high strengths, less shrinkage, improved durability and lower cost per functional unit [38].

## 4. Material Investigations

### 4.1. Hypothesis and Concept of Investigations

The presented investigations pursue the goal to investigate the LP3DCP process fundamentally on a material level. With this technique, it is aimed to produce elements with high compressive strengths while introducing coarse aggregates and herewith significantly reduce the CO_2_ footprint. It is necessary that the sprayed fine grain concrete completely penetrates the individual layer of the particle bed in order to provide a mechanical bonding in between the layers. Consequently, it is of crucial importance to control the fine grain concrete penetration in each layer, as it is also reported in the literature. From this, the main parameters for the experimental investigations are deduced.

In Lowke et al. [10] and Pierre et al. [35], it was shown that the compressive strength of 3D printed components fabricated with the SPI method is related to the penetration depth of the cement paste. Mechanical test results revealed that the voids of each layer need to be filled entirely in order to ensure a good cohesion of the component and herewith enable a high compressive strength. According to the theoretical framework in Pierre et al. [35], it was possible to compute a maximum yield stress value for which a full layer penetration was possible with the SPI technique. Although this framework was developed for the static pressure of the liquid, it can be assumed that the underlying relationship between the rheological parameters of the applied liquid and the properties of the particle bed are qualitatively comparable to the boundary conditions (where material is applied with high kinetic energy) at hand. Therefore, the same general assumptions can be made for the penetration depth $h_{pen}$ of a non-Newtonian fluid with yield stress $\tau_{c}$ into a particle bed:

(1) $$h_{pen}\sim \frac{1}{\tau_{c}}$$

As such, a lower yield stress of the liquid is accompanied with a larger penetration depth of the liquid into the particle bed. However, in the shotcrete process, a high yield stress may be counterbalanced with the high kinetic energy induced by the application process, and herewith a full penetration of the layer may occur. The high kinetic energy of the sprayed material most probably helps mitigate the risk of bad interlocking in between single layers via a higher penetration depth than can be expected when solely considering the yield stress of the material expected according to Equation (1). If this is prevalent, higher yield stresses are desirable since a higher geometric precision is targeted.

Additionally, the maximum value of the intruding material’s yield stress $\tau_{c,max}$ that allows a complete filling of an aggregate layer is dependent on the packing density $\varphi_{s}$ of the aggregates:

(2) $$\tau_{c,max}\sim \frac{1-\varphi_{s}}{\varphi_{s}}$$

With increasing packing density, the maximum yield stress of the fluid needs to be lower in order to enable a full penetration of the layer.

It needs to be emphasized that the chosen process parameters in the shotcrete process will most probably have an effect on the penetration depth of the fluid. Here, the nozzle-to-strand-distance, the volume air flow induced at the nozzle, the concrete discharge rate and the traverse speed of the nozzle are parameters which have an effect on the strand properties in Shotcrete 3D Printing [20] and are therefore expected to have an effect on the penetration depth in LP3DCP as well. The process parameters are strongly linked to the kinetic energy of the sprayed material. As mentioned, the high kinetic energy related to this shotcrete process is considered to be beneficial for the interlocking in between single layers. An unwanted displacement of particles does not occur. However, in the presented investigations, the process parameters are kept constant, and herewith the effects of process parameters are not discussed extensively. Based on these assumptions, two approaches are considered for controlling the fine grain concrete intrusion into the large particle bed:

- Modifying the rheological properties (especially the yield stress) with the help of superplasticizers and stabilizers. On the one hand, a low yield stress is required to enable a full penetration of the aggregate layer by means of the injected material. On the other hand, the risk of the injected material running through the particle bed layer is increased, i.e., the geometric precision is reduced.
- Modifying the packing density of the large particle bed. A higher packing density may have the advantage of a better mechanical performance, since the increase in aggregate volume is commonly known to increase the compressive strength of concrete, e.g., [41]. However, the penetration depth of the applied material is reduced and may hinder a good mechanical bonding between the layers, which finally affects the mechanical performance in a negative way.

In order to understand the basic mechanisms of the LP3DCP technology better, a systematic investigation is carried out. The rheological behavior of sprayed concrete mixtures—varying in stabilizer and superplasticizer content—is determined. The material is sprayed onto two different particle beds of different particle size distributions, and the resulting LP3DCP strands are mechanically and structurally evaluated. Finally, the best material combination is used in order to produce a large-scale demonstrator (compare with Section 5).

### 4.2. Materials

#### 4.2.1. Particle Bed

The dry particle bed consists of recycled coarse aggregates with a density of 1620 kg/m^3^. Particle bed A is sieved to contain aggregates in only one particle size group, i.e., where the largest particle has at most twice the diameter of the smallest particle (d_max_ ≤ 2 d_min_, here: 16–32 mm); compare with [42]. Particle bed B is a binary packing, consisting of two single particle size groups with a size ratio of 1:4 (here: 4–8 mm and 16–32 mm). These particle size groups are mixed at a volume ratio of V_4–8_/V_16–32_ = 0.27 since a packing density maximum is expected for this composition of particle size groups [43,44].

For particle bed A, a packing density of 0.41, and for particle bed B, a higher packing density of 0.44, is reached in the formwork; compare in Table 1.

#### 4.2.2. Fine Grained Sprayed Concrete

The material, which is sprayed onto the particle bed, consists of ordinary Portland cement (OPC, CEM I 52.5 R) and quartz sand with a maximum grain size of 3.15 mm. A detailed overview of all used components and chemical admixtures is given in Table 2. In total, six mixtures are studied containing 0.0, 0.05 or 0.1% by weight of cement (bwoc) stabilizer and either 0.4 or 0.5% bwoc superplasticizer. The names of the materials are chosen according to their stabilizer (ST) and superplasticizer (SP) content. e.g., ST0.0_SP0.4 contains 0.0% bwoc of stabilizer and 0.4% bwoc of PCE superplasticizer.

### 4.3. Methods

#### 4.3.1. Rheology of the Fine Grained Sprayed Concrete

The mixing regime for the rheological experiments is designed to mimic the rheological properties of the material which is produced in the large mixer of the printing experiments (compare in Section 5). This was evaluated in pre-tests for the used mixing volume of 4 L.

The following mixing regime is applied to the material, which was stored at 20 °C: 

I. The cement, ground limestone and—if applicable—stabilizer are placed in the mixer. From 0:00 to 0:15 min, water, and from 0:15 to 1:00 min, aggregates, are added to the mixer (EL 5 profi, Eirich, Hardheim, Germany). The mixer is equipped with a star-type rotor, which is rotating with 125 s^−1^. The mixing pan rotates with 45 s^−1^. After a total mixing time of 1:00 min, the superplasticizer is added.

II. Continuous mixing for a further 3:00 min is conducted.

After mixing, the material is put into the rheometer and is presheared for 10 s with a 4-bladed stirrer. Then, 6:30 min after the water addition, the rheometer is started. For the rheological characterization of the used shotcrete material, the rheometer Viskomat XL (Schleibinger, Buchbach, Germany) is used with a 6-bladed vane paddle in a cup (vane height *h_vane_* = 70 mm, vane radius *r_vane_* = 35 mm, cylinder radius r_cyl_ = 67.5 mm). A total volume of 3 liters of material is tested.

**Figure 2:**
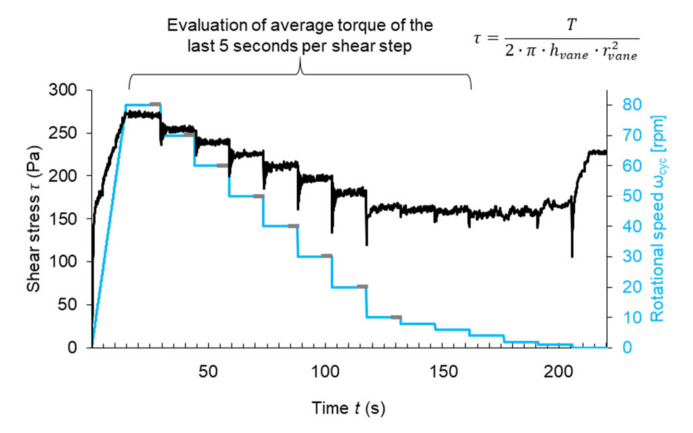
Shear protocol in the rheometer and exemplified shear stress measurement of ST0.0_SP 0.4%.

A rotational velocity-controlled rheometer profile defined by a sequence of rotational speeds is used for the material characterization. The profile consists of a linear increase in rotational speed and then a stepwise decrease in rotational speed, whereby each step has a duration of 15 s. For the evaluation of the material’s rheological properties, the data of the first eight downward ramps are used for a Bingham evaluation. For the evaluation the torque *T* (Nm), values of the last five seconds of each rotational step are averaged. In Figure 2, the shear profile and an exemplified measurement of ST0.0_SP 0.4% are shown. Finally, the Reiner–Riwlin equation is used to calculate the Bingham model parameters (plastic viscosity and yield stress) [45,46].

#### 4.3.2. Large Particle 3D Concrete Printing for Material Investigations

A small lab-scale unit, namely the Smart Additive Manufacturing Material Investigator (SAMMI), is used to spray the material onto the particle bed. In order to process the shotcrete, SAMMI contains an x-z-linear axis with a maximum gantry speed of up to 5.0 m/min. The shotcrete material is produced with a compulsory mixer and pumped (Mader WM Variojet FU) through a 5 m long hose with an inner diameter of 35 mm to a shotcrete nozzle, where the concrete is sprayed by pressurized air.

For the experiments at hand, the discharge rate of the concrete pump is set to $\dot{Q_{concrete\;}}$ = 0.8 m^3^/h; the working distance from the nozzle to the particle bed surface is 200 mm; the volume air flow induced in the nozzle is 45 m^3^/h.

The gantry speed is adjusted in order at least to fill the voids in the particle bed, i.e.,:

(3) $$v_{gantry}=\frac{\dot{Q_{concrete}}}{b\;d_{lay}\;\left(1-\varphi_{s}\right)}$$

with b being the width of the applied strand (assumed 16.5cm), $d_{lay}$ being the layer height and $\varphi_{s}$ being the packing density of the powder bed. This results in gantry speeds ranging from 4.3 m/min (particle bed A) and 4.5 m/min (particle bed B).

After manufacturing, all specimens are stored at 20 °C and 65% relative humidity until the determination of the mechanical properties.

For the large particle experiments, batches of 55 L are mixed in a compulsory mixer (Mader WM Jetmix 125/180). Therefore, water is added to the mixer. In the first 2:00 min of mixing, all dry components are continuously added to the mixing container while the mixing tool rotates. Firstly, a mixture of cement, ground limestone and stabilizer is added. Secondly, the premixed aggregates are added. The superplasticizer is added constantly over the entire 2:00 min. Afterwards, the batch is continuously mixed for a further 2:00 min.

For providing the particle bed, a building box with a base area of 200 × 1000 mm^2^ is used. In this building box, 32 mm high particle bed layers are applied and evenly distributed by a rake. The shotcrete material is then applied onto the particle bed with a nozzle distance of 200 mm. After the application of the sprayed concrete, the next 32 mm high particle bed layer is applied. The walls of the building box consist of three separate 64 mm high modules, which allow the building box to be raised during the printing process. Thus, two layers can be printed per wall module. A total of 6 layers are printed per test specimen.

#### 4.3.3. Compressive Strength, Geometric Precision and Inner Structure

**Figure 3:**
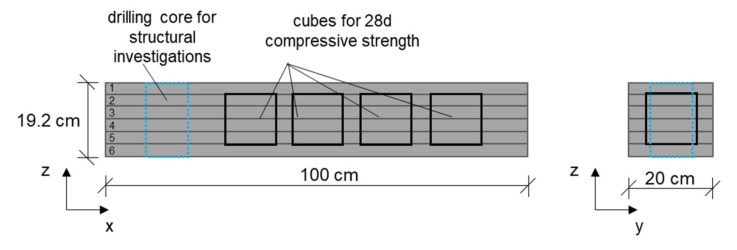
Principle of sampling in the material study.

An overview of the principle of the sampling is shown in Figure 3. Specimens are designated in order to evaluate the mechanical performance on cubes as well as the inner structure of the produced strands.

The geometry of the strands is determined after excavation from the building chamber with a ruler at two positions.

To investigate the inner structure qualitatively with regard to the bonding between two layers, a drilling core (diameter 100 mm) is taken from the produced particle bed strands. The specimen is placed into a µCT and 3D scanned (GE phoenix, voltage: 160 kV, current: 240 µA, number of images: 1000, image average: 3, filter: 0.1 mm Cu, exposure: 500 ms for all specimens, voxel edge length: 131 µm, multiscan with 4 sections; total scan time: 2 h 13 min). From the X-ray projections of each drilling core, the volumetric image is obtained by applying a 3D reconstruction algorithm with the software phoenix datos|x2 (GE Sensing & Inspection Technologies, Boston, MA, USA). Within the software VG studiomax 2.2 (Volume Graphics, Heidelberg, Germany), the drilling cores are further analyzed regarding their porosity. The evaluated volume of interest is defined from the middle of layer 1 to the middle of layer 3. This volume is segmented into solids and voids in order to determine the air void content within the drilling core. In addition, photos are taken for visual inspection.

In order to examine mechanical properties of the specimens taking into account the isotropy, four cubes with a side length of 10 cm are cut perpendicular to the layer direction. The compressive strength of the cubes is tested after 28 days of standardized storage according to DIN EN 12390-3:2019-10. Two cubes are tested by applying the load in parallel, and two cubes are tested perpendicular to the layer direction. In order to obtain a comparison to the sprayed material, monolithic cubes for all stabilizers and SP-dosage-materials were produced without spraying. Therefore, the volume of air flow is reduced to 0 m^3^/h, and the nozzle is removed during the tests. Then, the material is cast into prism moulds and compacted for 10 s on a vibration table. The conventionally produced samples are stored together with the printed specimens under standardized conditions (20 °C, 65% r.H.) and are tested after 28 days.

### 4.4. Results

#### 4.4.1. Rheological Properties of the Fine Grained Sprayed Concrete

**Figure 4:**
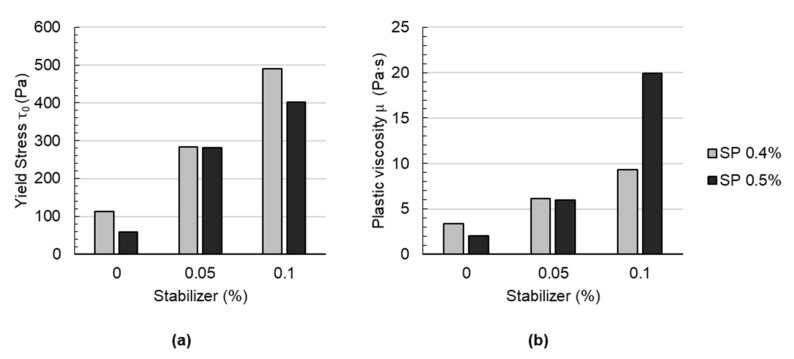
Rheological parameters of the sprayed concrete: (**a**) yield stress and (**b**) plastic viscosity of mortars containing various amounts of stabilizer and superplasticizer.

In Figure 4, the yield stress and plastic viscosity of mortars containing various amounts of stabilizer (0, 0.05% and 0.1% bwoc) and superplasticizer (0.4 and 0.5% bwoc) are shown. Independent of the superplasticizer dosage, yield stress and plastic viscosity increase with an increasing stabilizer dosage. This was expected since the cohesiveness and stability of cement-based materials are known to be enhanced with viscosity-modifying agents such as stabilizers [47]. This principle of operation is derived from various physico-chemical phenomena [48,49]: (a) water retention, which increases the viscosity of the liquid phase, (b) the formation of a gel and polymer entanglement, which blocks the mobility of water, (c) particle polymer interaction leading to particle-particle bridging and thus entrapped water. Finally, these mechanisms are responsible for building yield stress and viscosity in the suspension. With an increasing stabilizer dosage, the effect is considered to be more pronounced [50,51]. It is worth mentioning that, unexpectedly, for mixes with 0.1% stabilizer, significantly higher plastic viscosities are observed at a higher superplasticizer dosage (ST0.1_SP0.5) than for a lower superplasticizer dosage (ST0.1_SP0.4); see Figure 4, right. This effect is not observed at lower stabilizer contents, i.e., 0.05% or 0%. This might be due to counterproductive, i.e., antagonistic, effects, which occur when the stabilizer and superplasticizer interact [52].

#### 4.4.2. Geometry of the Produced Strands

**Figure 5:**
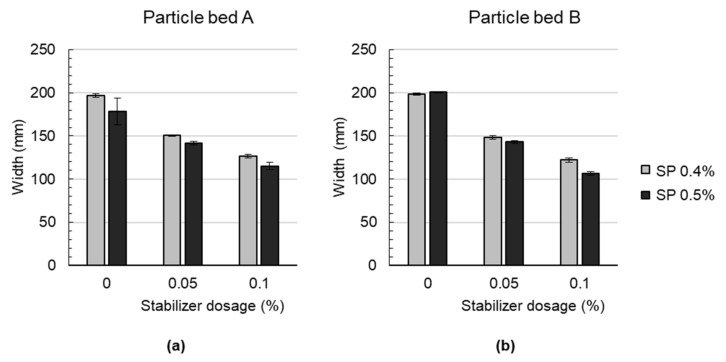
Width of the LP3DCP strands (**a**) with particle bed A and (**b**) particle bed B.

A decrease in strand width is observed for particle bed A and B when the stabilizer dosage is increased; see Figure 5. Compared to the effect of the stabilizer dosage, the effect of various dosages of the superplasticizer is negligible. The mean width of the strands is with 151.5 mm, which is slightly lower for particle bed A than for particle bed B with 153.2 mm. It may occur that the shotcrete material tends to penetrate the particle bed A more in a vertical direction than in particle bed B. In particle bed B, the resistance for vertical penetration is higher due to the higher packing density. Due to a higher penetration resistance in the vertical direction, the material may tend to distribute more in a horizontal direction on top of the particle bed.

**Figure 6:**
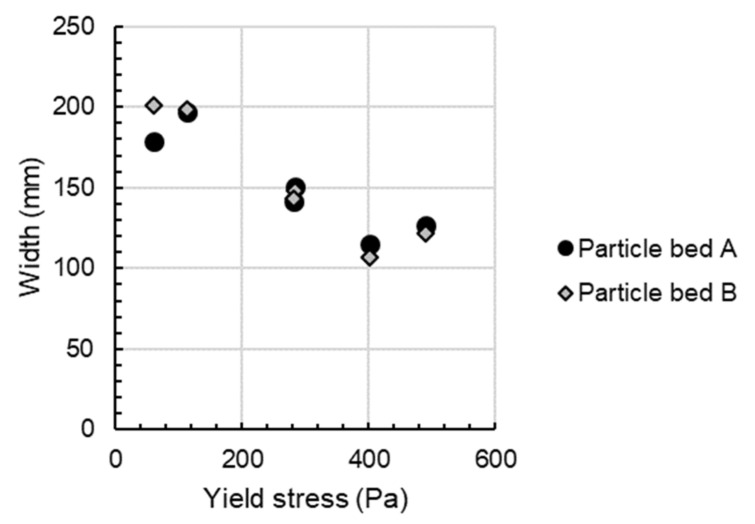
An increase in yield stress correlates with a decrease in strand width for particle bed A and B.

Additionally, it can be stated that the width of the strand is highly dependent on the rheological properties of the material. In Figure 6, it is shown that an increase in the shotcrete’s yield stress correlates with a decrease in the width of the strand. For example, when the yield stress of the material is increased from 59.6 Pa to 490.7 Pa, the strand width decreases from 200 mm to 122 mm for particle bed B.

#### 4.4.3. Structural Bonding Characteristics

**Figure 7:**
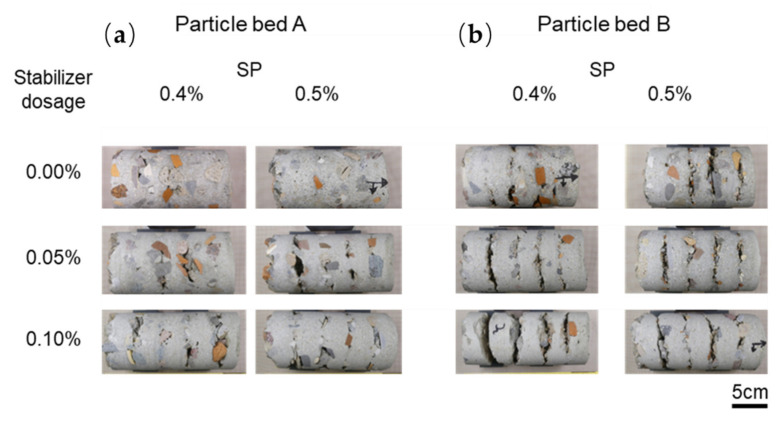
LC3DCP drilling cores with 5 layers from particle bed A (**a**) and B (**b**).

Drilling cores are inspected visually; see Figure 7. It is observed that the layers reveal fewer voids and a better interlayer bonding when particle bed A is used instead of particle bed B. Additionally, there is a tendency towards more voids in particle bed B, and thus towards poorer interlayer bonding.

**Figure 8:**
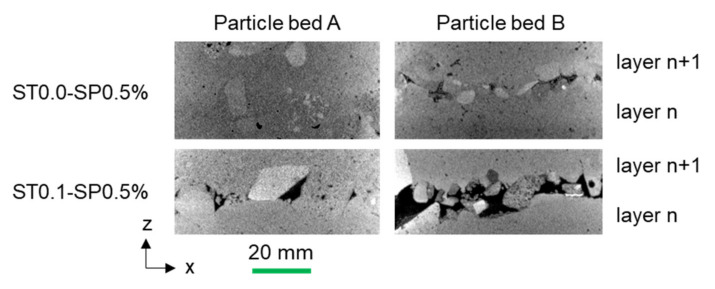
Exemplary µCT investigations of the interlayer in between two strands of particle bed A (**left**) and particle bed B (**right**) with 0% (**top**) and 0.1% (**bottom**) stabilizer and 0.5% SP. Higher density appears white; lower density appears black.

In Figure 8, µCT investigations of the area in between two layers for specimens without the stabilizer (ST0.0–SP0.5) and with a high stabilizer dosage (ST0.1_SP0.5) are shown for particle bed A and B. The tendency of a better structural bonding (and fewer voids) in between two layers when less stabilizer is used, as observed before on the outside of the drilling cores, is also evident here. Moreover, particle bed A is favorable compared to particle bed B since voids are reduced, and thus there is better interlayer bonding.

**Figure 9:**
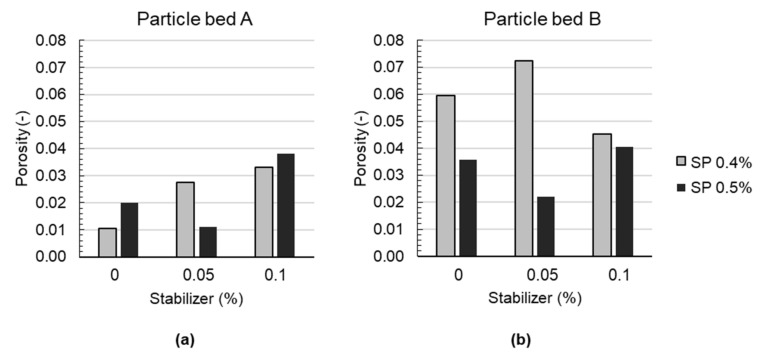
Results of porosity determination with µCT of drilling cores from LP3DCP specimens for (**a**) particle bed A and (**b**) particle bed B.

Additionally, the porosity of the three layers is determined with µCT. The obtained results for the porosity in particle bed A and B are shown in Figure 9 for all investigated mixes. It is observed that particle bed A reveals, with approx. 1–4 V = vol.%, significantly lower porosity values than particle bed B, with approx. 2–7 vol.%. For particle bed A, an increase in the stabilizer dosage is accompanied with an increase in porosity. This is to be expected, as an increase in the stabilizer dosage is accompanied by an increase in yield stress (Figure 4a), which is associated with a reduced penetration depth of the binding material and thus an increase in (local) porosity. For particle bed B, the results are not as clear. In general, a worse structural bonding is observed for particle bed B (compare Figure 7 and Figure 8). Due to the higher packing density in particle bed B, it can be stated that the yield stress of the applied material has to be lower than for the material applied on particle bed A in order to enable full penetration of the layer (compare with Equation (1)).Therefore, it is assumed that the binding material may be too coarse and cohesive to flow freely into the comparably small pore space of particle bed B. Additionally, a higher tortuosity and surface appears with higher packing density, which is accompanied with a higher frictional area between the binding material and particle. As a consequence, the binding material does not reach a high penetration depth, and it remains on top of the particles. Even a very flowable material, without the usage of any stabilizer, does not lead to a good coupling in between two layers, as it can be observed in the higher number of voids in particle bed B compared to particle bed A for ST0.0–SP0.4 and ST0.0_SP0.5.

#### 4.4.4. Compressive Strength

**Figure 10:**
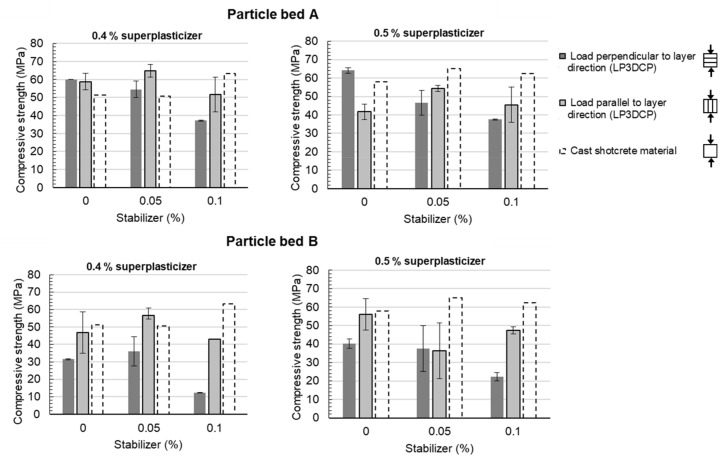
Compressive strength of LP3DCP specimens for particle bed A (**top**) and B (**bottom**), superplasticizer dosage 0.4% (**left**) and 0.5% (**right**), load direction perpendicular (dark grey bars) and parallel (light grey bars) to the layer orientation as well as compressive strength of the pure shotcrete material without coarse recycled aggregates conventionally cast in moulds (dashed bars).

The mechanical characterization of the specimens, based on compressive strength tests after 28 days, is shown in Figure 10. The diagrams presented show the results of printed specimens for particle bed A (upper diagrams) and B (lower diagrams), for the superplasticizer dosage 0.4 (left) and 0.5% (right) and for a load application perpendicular (dark grey bars) and parallel to the layer orientation (light grey bars). Furthermore, compressive strength values of the pure shotcrete material, i.e., without particle bed aggregates, cast in moulds without pressurized air are displayed as dashed bars.

For particle bed A with a load application perpendicular to the layer direction (dark grey bars), a reduction in compressive strength can be observed by the increase in the stabilizer dosage for both a 0.4% and 0.5% superplasticizer dosage. Thus, the highest compressive strength values are measured for 0% stabilizer at 60.0 MPa for ST0.0–SP0.4 and 64.1 MPa for ST0.0–SP0.5, respectively. For the pure shotcrete material without particle bed aggregates, compressive strengths between 51.2 MPa (ST0.0–SP0.4) and 57.9 MPa (ST0.0–SP0.5) could be determined. They are thus slightly below the compressive strengths of the printed specimens.

Similarly, for specimens produced in particle bed B (lower diagrams), a reduction in compressive strength perpendicular to the layer direction can be observed with an increase in the stabilizer dosage. However, these values are significantly lower than those of the pure shotcrete material. Here, the maximum compressive strength is reached for ST0.0–SP 0.5% with 40.3 MPa. The lowest compressive strengths were measured in the samples, which were produced in particle bed B with ST0.1_SP 0.4% tested perpendicular to the layer direction. Here, an average compressive strength of 12.3 MPa could be achieved.

**Figure 11:**
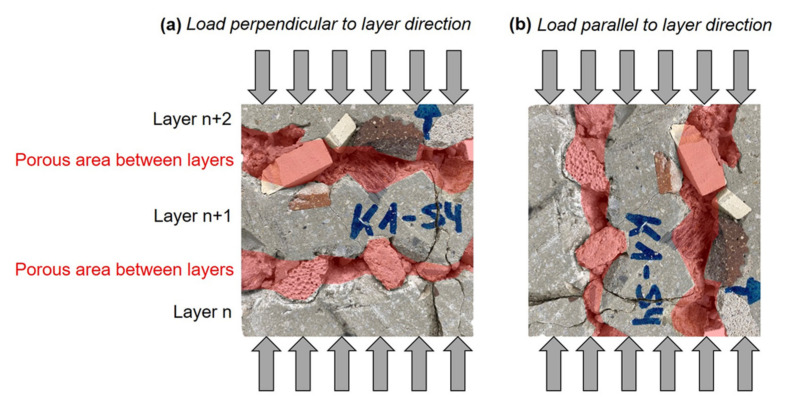
Layer orientation in compressive strength test specimens (particle bed A, ST0.1_SP0.5) for (**a**) load perpendicular to layer orientation and (**b**) load parallel to layer orientation—porous areas between layers are marked in red.

It can be noticed that the specimens, which were tested parallel to the layer direction (light grey bars), show higher standard deviations but also higher compressive strengths than those tested perpendicular to the layer direction, especially in combination with particle bed B and high stabilizer dosages. This could be explained by the serial and parallel connection of porous areas, i.e., defects between the layers; see Figure 11. For the mechanical tests perpendicular to the layer direction, the collapse of cavities in the areas between the layers may cause a mechanical failure of the entire specimen; see Figure 11a. The ability of the test specimen to absorb forces after an initial load collapse was not recorded due to the test machine’s break-off criterion. In the load case parallel to the layer direction, the forces may be transferred through the coherent shotcrete material layers; see Figure 11b. This could result in a concentrated load transfer through the applied shotcrete material, which leads to an increase in compressive strength compared to the 90° rotated layer alignment.

The results of the compressive strength tests demonstrated that specimens with a homogeneous material matrix, where a good penetration of the applied shotcrete material happened, were able to achieve higher compressive strength values than the pure shotcrete material (e.g., ST0.0–SP0.4). In contrast, such specimens, which are characterized by an inhomogeneous material matrix, showed a significant strength reduction compared to the compressive strength of pure shotcrete material as well as pronounced anisotropies caused by the inclusion of voids in between the layers.

On the basis of all results, particle bed A appears to be more suitable than particle bed B for LP3DCP. This is concluded from the good mechanical results with low scattering, especially for stabilizer dosages of less than 0.1%, as well as low porosities in between the layers. For the production of the large-scale demonstrator, the material ST0.05_SP0.4 reveals the best properties—a low porosity, very high mechanical strength while enabling control of the width of the strand. Therefore, this material is used for the production of the large-scale demonstrator.

### 4.5. Life Cycle Assessment of Large Particle 3D Concrete Printing

In order to check the potential of the LP3DCP technique compared to “regular” 3D printing techniques, an impact assessment of the functional unit 1m^3^ for (i) 3D printing with sprayed concrete only (without coarse aggregates), (ii) LP3DCP with naturally coarse aggregates ($\varphi_{s}$ = 0.41) and (iii) LP3DCP with recycled coarse aggregates ($\varphi_{s}$ = 0.41) is performed. The life cycle assessment encompasses the magnitude of potential and environmental impacts for the components of a functional unit with the cradle-to-gate-approach, i.e., production only.

Information for the ecological evaluation of the functional unit is taken from Ökobaudat [53], which depicts the country-specific situation in Germany, and the environmental product declaration (EPD)-online tool [54], which provides a standardized and verified database for the ecological evaluation of products and processes in accordance with ISO 14025:2010. In order to calculate the impact of the functional unit, the single components are considered according to their mass-based fraction (compare Table 2). For LP3DCP, also natural and recycled coarse aggregates are incorporated into the functional unit. Life cycle assessment indicators are considered for the life cycle modules in accordance with EN 15804. Therefore, the global warming potential as well as the acidification potential are investigated as factors having an effect on the global environment. Moreover, the total non-renewable primary energy requirement is considered as a factor for the resource use.

It is the intention to check whether LP3DCP can be a green and viable solution and not provide an extensive life cycle assessment. Therefore, the factors are limited to the most commonly known ones in order to work out the potential of LP3DCP.

**Figure 12:**
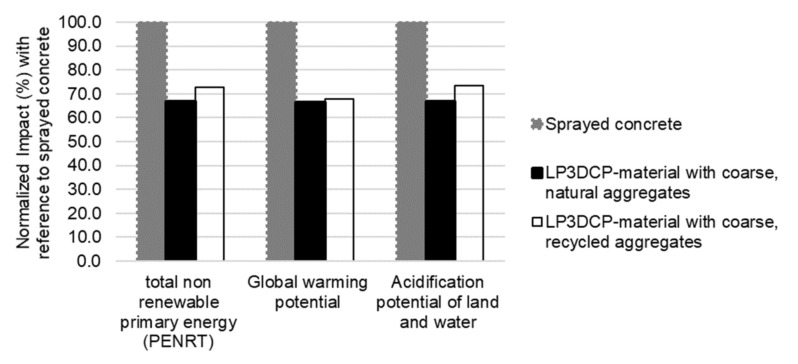
Ecological potential of LP3DCP compared to purely sprayed concrete shown for selected factors of a life cycle assessment.

The considered products for the life cycle assessment of a cradle-to-gate-approach encompass the cement (CEM I 52.5R), ground limestone, dried sand, water, superplasticizer and—for the LP3DCP-case—natural and recycled coarse aggregates. The absolute values of the considered life cycle assessment indicators are shown in Table 3. The normalized values are illustrated in Figure 12. It is shown that LP3DCP with natural or recycled coarse aggregates demands approximately 30% less non-renewable primary energy in comparison with the purely sprayed concrete. Moreover, the global warming potential and acidification potential of LP3DCP are approximately 30% lower than for the purely sprayed concrete, making the LP3DCP an eco-friendly additive manufacturing technique. It is worth noting that the results for the LP3DCP with naturally coarse aggregates and the recycled coarse aggregates are very similar. For the named indicators, this is a known effect [55]. Nevertheless, the main advantage of using recycled aggregates is the preservation of natural resources as well as the minimization of waste disposal.

## 5. Large-Scale Demonstrator

### 5.1. Experimental Setup

#### 5.1.1. Robotic Setup

The large-scale demonstrator is manufactured in the Digital Building Fabrication Laboratory (DBFL), a large robotic fabrication facility that allows for the performance of various additive and subtractive building processes via two digital-controlled portals [17]. For additive manufacturing processes, a 6-axis Stäubli TX200 industrial robot is integrated on the first portal. For the LP3DCP experiments, a shotcrete nozzle, previously developed for the Shotcrete 3D Printing process, is used [17,56]. The second portal is equipped for subtractive processes and comprises a 5-axis water-cooled CNC-mill which is designed for natural stone processing. This portal is used for the surface finishing of 3D-printed structures, either directly after printing when the concrete has not yet fully cured [57], or for precise cutting or milling in the cured state of the concrete [58].

#### 5.1.2. Additional Materials: Glass Fiber Reinforcement

**Figure 13:**
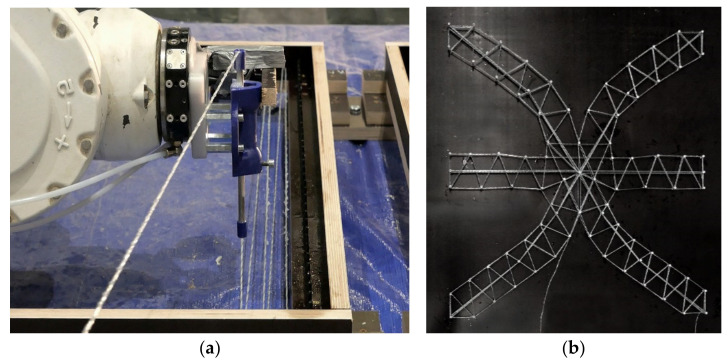
Dynamic fiber winding process for reinforcement: (**a**) winding principle around preplaced pins; (**b**) prefabricated fiber reinforcement inlay.

In addition to the materials described in Section 4.2, corrosion resistant glass fiber roving of type Advantex R25 HX14 2400 is used to produce individual reinforcement inlays. An epoxy resin coating protects the glass fiber from further deterioration in the concrete. In a custom-developed robot-based multi-directional dynamic fiber winding process, individual prefabricated reinforcement inlays are fabricated in an automated manner. For this purpose, the loose continuous filaments are pulled through a resin bath and wrapped with a helix structure before they are immediately winded around previously placed pins, Figure 13. This robotic reinforcement process was investigated for several additive manufacturing processes with concrete. While the technical details of this process are described in [59], a classification of this reinforcement strategy can be found in [60]. The cured reinforcement inlays are later placed between the particle bed layers at structurally relevant positions (see Section 5.3), and are then overprinted and hence structurally embedded in the object.

### 5.2. Design-to-Fabrication Workflow

#### 5.2.1. Form Finding

In order to assess LP3DCP’s new geometric degrees of freedom, a design studio was conducted at the department of architecture at Technische Universität Braunschweig. In this experimental design studio, preliminary design studies were developed and robotically fabricated on a 1:5 scale. For this, a process, similar to the Selective Paste Intrusion process [10,61,62] was implemented using lightweight UR 10 robots equipped with a customized extrusion print head.

**Figure 14:**
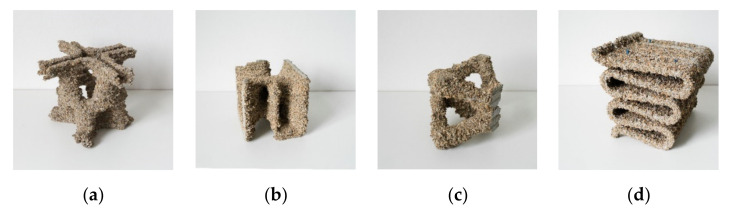
Design studies in scale 1:5 (25 × 25 × 25 cm^3^) conducted within the course “Digital Building Fabrication Studio” (DBFS) in winter semester 2020/2021 at Technische Universität Braunschweig, (**a**–**d**) various designs suggested for LP3DCP with (**b**) being the chosen design.

In terms of material, aggregates with a size of 6 mm and cement paste were used, whereas the designs produced in the studio comprised various digital designs and 3D modeling techniques (see Figure 14). Finally, a mathematical geometry inspired by the work of the Swedish sculptor Eva Hild and Carlo H. Séquins [63] was developed and chosen for fabrication on a 1:1 scale (see Figure 14b). Due to its complex geometry with a double curvature, numerous overhangs and numerous undercuts, this inspired geometry was found to be particularly suitable for the LP3DCP process. The underlying surface was generated by means of SubD modeling in Rhino 3D and designed to fit into a cubic formwork with an edge length of 1 m.

#### 5.2.2. Robotic Path Planning

For the path planning, a design-to-fabrication workflow is developed which involves slicing a given 3D geometry into horizontal layers corresponding to the thickness of the layer, i.e., here equivalent to the particle diameter; see Figure 1. Since the spraying end effector does not include a precise start-stop mechanism, the geometry is chosen so that the start and stop of the spray jet take place outside the particle bed. Accordingly, the robot path is created to have the end and start point of each layer 15 cm outside the formwork. The closing and opening routines of the integrated pneumatic pinch valve are programmed with a delay of 1.5 s, ensuring a full jet upon entry into the particle bed. Accordingly, the routine is reversed in time to stop the jet as soon as the print head moves out of the formwork. The waiting times between layers, which are necessary to place the new layer of large particles, are defined as user-defined pauses in which the process is continued after a button is pressed. For the fabrication of the 1:1 prototype, a wall thickness is chosen according to the width of the spray jet, which is estimated based on the results in Section 4.4.2.

### 5.3. Fabrication Process

#### 5.3.1. Additive Manufacturing

**Figure 15:**
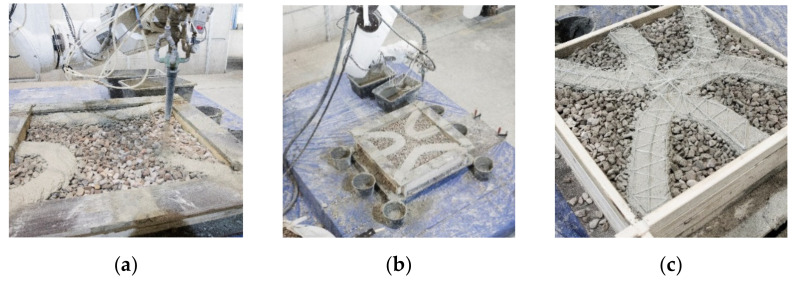
LP3DCP Additive Manufacturing process: (**a**) Close-up of the printing process during which the fine grain concrete is sprayed onto the particle bed; (**b**) formwork with printed path; (**c**) manually placed, prefabricated fiber-reinforcement.

For the additive manufacturing of the demonstrator, the first layer of the climbing formwork is mounted on a pallet on the clamping table inside the DBFL. The first layer of particles is placed manually using a shovel and is subsequently distributed evenly in the particle bed with a rake. Buckets are placed at the exit points of the robot path to collect excess material. After the first two layers are printed in about 80 s each (Figure 15a,b), the protective frame is lifted off; new formwork parts are stacked on top; finally, another layer of particles is applied and distributed. This process is repeated until layer 12, where the three contour lines meet in the middle to form one continuous contour. At this point, the prefabricated glass fiber reinforcement is inserted (see Figure 15c). A second prefabricated reinforcement structure is placed at layer 26, in which a second continuous contour is formed.

#### 5.3.2. Demoulding and Edge Milling

**Figure 16:**
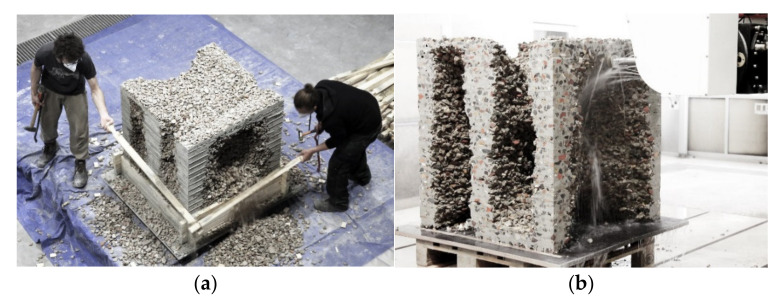
Demoulding and post-processing: (**a**) dismantling of the stackable formwork elements; (**b**) milling of the edges.

After 24 h, the final demonstrator is exposed with the help of small shovels. The unbound aggregates are removed and can be reused for the manufacturing of another object (Figure 16a).

**Figure 17:**
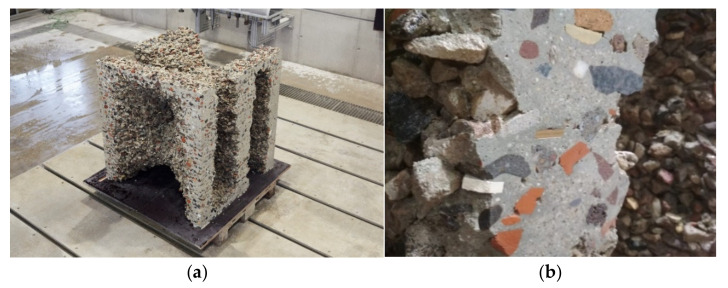
Final demonstrator object: (**a**) overall geometry with milled edges; (**b**) close-up of the milled edges.

Due to the adopted path planning, in which the robot moves beyond the edge of the formwork, visible imprints of the formwork are created on the edges of the object (Figure 16a). The surfaces are post-processed both in terms of visual quality and from a functional point of view, e.g., with regard to the creation of precise dry joints. For this purpose, the upper 15 mm of the edge surfaces are milled off with the second portal. Using a water-cooled 91 mm milling bit, a cutting depth of 5 mm and a feed rate of 500 mm/min at 2500 rpm spindle speed, a terazzo-like surface is milled (Figure 16b), clearly exposing the aggregates in the cut surface (Figure 16b and Figure 17b).

### 5.4. Results

The production of a single layer, with the individual steps of adding the next formwork layer, placing the particles manually, distributing the particles evenly and spraying on the fine grained concrete, consumed an average of 4 min, with two persons involved. It is evident that the printing itself took the least time (80 s), while the particle placement took the most time (160 s). In total, the production time for the additive manufacturing process of the object was 128 min, whereas the milling of the edges consumed another 240 min.

As expected from the fundamental material investigations described in Section 4, the milling of the edges unveiled a good penetration of the sprayed concrete through the particle layers, with no visible separation of the layers.

## 6. Summary

In this paper, a new additive manufacturing process, called Large Particle 3D Concrete Printing (LP3DCP), was introduced with fundamental material investigations and the production of a demonstrator. In this novel approach, large particles, i.e., coarse aggregates, serve as a particle bed, and material is applied via a robotically controlled shotcrete process. The use of coarse aggregates reduces the cement volume fraction by more than 50% compared to structures conventionally printed with mortar. Thus, the ecological footprint of the process can be significantly reduced. For the investigations presented in this paper, recycled aggregates were used, which further preserves natural resources.

First, a fundamental material investigation was performed in order to derive a beneficial combination of particle bed and applied shotcrete material. Two particle beds varying in their composition with a maximum aggregate size of 32 mm were used. The rheological behavior of the shotcrete material was systematically varied by adding three dosages of the stabilizer and two dosages of the superplasticizer. It was shown that an increase in yield stress of the applied shotcrete material on both particle beds led to lower strand widths. An increase in yield stress also reduced the penetration depth of the applied shotcrete material in the particle bed and thus decreased the interlayer bonding quality. Moreover, it was shown that the particle bed consisting of one particle size group revealed significantly better interlayer bonding and lower porosity than the particle bed consisting of two particle size groups. The enhanced bond quality was accompanied by higher mechanical strengths of the printed specimens. In addition, it was shown that the LP3DCP allows for the reduction in the cement content while slightly improving the compressive strength of the structure compared to the purely shotcrete material. In a life cycle assessment of the LC3DP process, it was shown that the global warming potential and acidification potential, as well as the use of total non-renewable primary energy, are reduced by more than 30% compared to a 3D printing process using the purely shotcrete material without coarse aggregates.

Afterwards, the production of the demonstrator was executed with the material combination that revealed the best results in terms of mechanical strength, interlayer bonding and geometry in the fundamental material investigation. A first concept for the integration of tensile reinforcement was presented as well. It can be stated that the LP3DCP technique offers extended degrees of geometric freedom and allows for the production of overhangs, cantilevers and intersections that would otherwise not be possible with additive manufacturing methods such as extrusion. Lastly, the surface finishing of the edges using subtractive milling allows for the production of precise visual and functional surfaces, and offers a good contrast to the otherwise rough surfaces delivered with the LP3DCP process. This reveals great potential for future applications.

## 7. Outlook

In addition to the benefits summarized above, there is further potential to improve the LP3DCP process. Firstly, a higher degree of automation should be aimed for in the future: in particular, the placement and even distribution of the particles, which consume a greater part of the production time, should be automated. In that regard, a second robot could be used to place the material automatically. In addition to the automation of the particle placement and distribution, there is significant potential for increasing efficiency through the automated installation of the formwork. For this purpose, small-scale particle bed printing systems already provide solutions in which the formwork does not grow along with the structure, but the particle bed is instead lowered automatically with each printed layer. Moreover, also with regard to automation, a more precise start-stop mechanism of the spraying jet would significantly increase the degree of geometric freedom, as the printed paths could start and stop in the middle of the particle bed. In particular, this would open new avenues for structural component improvements through topology optimization. With regards to the structural performance, the integration of reinforcement needs to be developed further. In this first demonstrator, only horizontal reinforcement was integrated, whereas further investigations must also address reinforcements perpendicular to the layer orientation. For this, a suitable combination would be with the Wire Arc Additive Manufacturing process, in which the reinforcement would be built up simultaneously layer by layer using droplets of steel [64].

A second area for improvement is the use of more ecologically sprayed concrete. This can be achieved by, for example, reducing the cement content, replacing the cement clinker with supplementary materials or implementing the total replacement of Portland cement with alkali-activated binders [55].

## Figures and Tables

**Table 1:** Characteristics of the particle beds.

Components	Particle Bed A	Particle Bed B
Particle Size (mm)	16–32	4–32
Bulk density (kg/m^3^)	661.5	706.3
Aggregate density (kg/m^3^)	1620	1620
Packing density (-)	0.41	0.44

**Table 2:** Mixture composition of the fine grained sprayed concrete.

Components	Value	Unit
Portland Cement (CEM I 52.5 R)	600	kg/m^3^
Ground limestone	97	kg/m^3^
Aggregate, d = 0–3.15 mm	1258	kg/m^3^
Water	270	kg/m^3^
Stabilizer	0.0, 0.05 and 0.1	% bwoc
PCE superplasticizer	0.4 and 0.5	% bwoc

**Table 3:** Life cycle assessment indicators for 1m^3^ of a regular 3D printing material and LP3DP.

Life Cycle Assessment Indicator Related to 1m^3^ 3D-Printed Concrete	Sprayed Concrete	LP3DCP with Naturally Coarse Aggregates	LP3DCP with Recycled Coarse Aggregates
Total non-renewable primary energy (MJ/m^3^)	2658.0	1765.6	1936.2
Global warming potential (kg CO_2_-Eq./m^3^)	680.5	452.0	461.4
Acidification potential of land and water (kg SO_2_-Eq./m^3^)	0.5	0.4	0.4

## Data Availability

Not applicable.
